# Schooling experiences in children with long-gap esophageal atresia compared with children with esophageal atresia and primary anastomosis: a Swedish study

**DOI:** 10.1186/s13023-023-02846-8

**Published:** 2023-08-07

**Authors:** M. Dellenmark-Blom, C. Reilly, E. Öst, S. Örnö Ax, J. F. Svensson, A.-M. Kassa, L. Jönsson, K. Abrahamsson, V. Gatzinsky, AM. Tollne, E. Omling, P. Stenström, H. Engstrand Lilja

**Affiliations:** 1 Department of Pediatric Surgery, Queen Silvia Children’s Hospital, Sahlgrenska University Hospital, Gothenburg, Sweden; 2 Department of Pediatrics, Institute of Clinical Sciences, Queen Silvia Children’s Hospital, Gothenburg University, 416 85 Gothenburg, Sweden; 3 Department of Pediatric Surgery, Karolinska University Hospital, Stockholm, Sweden; 4 Department of Women’s and Children’s Health, Karolinska Institutet, Stockholm, Sweden; 5 Department of Pediatric Surgery, University Children’s Hospital, Uppsala, Sweden; 6 Department of Women’s and Children’s Health, Uppsala University, Uppsala, Sweden; 7 Department of Pediatrics, Clinical Sciences, Lund University, Lund, Sweden; 8 Department of Pediatric Surgery, Skane University Hospital Lund, Lund, Sweden

**Keywords:** Esophageal atresia, Rare disease, Schooling experiences, School support, Special education mental health

## Abstract

**Background:**

Children with long-gap esophageal atresia (LGEA) risk living with aerodigestive morbidity and mental health difficulties. No previous study has investigated their experiences of schooling, despite the importance of schools in children’s development, learning and social relationships. We aimed to describe experiences of schooling in children with LGEA in Sweden in comparison with children with EA who had primary anastomosis.

**Method:**

Children with LGEA aged 3–17 were recruited nationwide in Sweden. One parent completed a survey on their child’s school-based supports (according to definitions from the Swedish National Agency for Education), school absence, school satisfaction, school functioning (PedsQL 4.0), mental health (Strength and Difficulties Questionnaire) and current symptomatology. School data were compared between 26 children with LGEA to that from 95 children with EA who had PA, a hypothesized milder affected group. Mental health level was determined using validated norms; abnormal ≥ 90 percentile. Data were analyzed using descriptives, correlation and Mann–Whitney-*U* test. Significance level was *p* < 0.05.

**Results:**

Formal school-based support was reported in 17 (65.4%) children with LGEA and concerned support with nutritional intake (60%), education (50%) and medical/special health needs (35%). The prevalence of school-based support was significantly higher compared to children with PA overall (36.8%, *p* = 0.013) and regarding nutritional intake support (20%, *p* < 0.001). In children with LGEA, school-based support was related to low birth weight (*p* = 0.036), young child age (*p* = 0.014), height ≤ −2SD for age/sex (*p* = 0.024) and an increased number of aerodigestive symptoms (*p* < 0.05). All children with LGEA who had abnormal mental health scores had school-based support, except for one child. Nine children with LGEA (36%) had school absence ≥ 1times/month the past year, more frequently because of colds/airway infections (*p* = 0.045) and GI-specific problems compared to PA (*p* = 0.003). School functioning scores were not significantly different from children with PA (*p* = 0.34) but correlated negatively with school-based support (< 0.001) and school absence (*p* = 0.002). One parent out of 26 reported their child’s school satisfaction as “not good”.

**Conclusions:**

Children with LGEA commonly receive school-based support, reflecting multifaceted daily needs and disease severity. School absence is frequent and related to poorer school functioning. Future research focusing on academic achievement in children with EA is needed.

**Supplementary Information:**

The online version contains supplementary material available at 10.1186/s13023-023-02846-8.

## Background

Esophageal Atresia (EA) is a rare congenital anomaly characterized by a discontinuity of the esophagus. In 10–15% of the cases, the gap between the two esophageal ends is too long to perform a primary anastomosis at the initial surgery, which is usually referred to as long-gap EA (LGEA) [1, 2]. LGEA can be managed by inserting a gastrostomy for enteral feeding, allowing for spontaneous growth of the esophageal segments, then performing a delayed primary anastomosis when the child is 3–4 months old [3]. Esophageal replacement may be applied using stomach, jejunum or colon and with the conduit of choice depending on the preference of the surgical center [1, 2]. Children with LGEA carry a high risk of future morbidity [3, 4]. They more commonly present with cardio-vascular malformations [5, 6], genetic disorders and prematurity/low birth weight as opposed to short-gap EA [5]. They face at higher risk of developing long-term complications, including dysphagia, gastroesophageal reflux disease, feeding difficulties and respiratory disease [4, 7–12].

Children spend a considerable amount of time in school and schools therefore can play an important role in their development, learning, social relationships, and growth as well as their mental health. According to the Convention on the Rights of the Child, each child has the right to an education that shall enable the child to develop his or her fullest potential [13]. In the UN resolution for persons living with a rare disease [14], it is clearly described that inclusive and equitable quality education and lifelong learning opportunities without discrimination are essential for the full, equal and meaningful participation in all aspects of life [14].

Previous studies have shown that 22–35% of children with EA are recipients of special education [15–17] and those with associated anomalies risk poorer school-functioning [18, 19]. No study has, however, particularly focused on children with LGEA. In a recent nationwide Swedish study [20], we found that 46% of children with LGEA had elevated levels of mental health difficulties according to their parents, especially peer relationship problems and difficulties with hyperactivity/inattention. Recommendations for care and treatment for patients with EA have been published by several expert stakeholders [21–23] including for LGEA [1, 2]. Yet, none of these provide advice on how to care for mental health needs or develop collaborative strategies between specialized health care centers and schools that accommodate for the needs of these children. The aim of this study was to describe the experiences of schooling in children with LGEA in Sweden, including prevalence, type and factors related to school-based supports and school absence as well as the level of school functioning and satisfaction.

## Material and methods

### Ethics

This study was approved by the Swedish Ethical Committee; 958–13, 2019–04930 and 2020–04310. Children with LGEA with need for school-based support were provided with such support, if not already in place.

#### Setting

In Sweden all children aged between 1 and 5 years have a guaranteed place at a preschool. The Swedish preschool includes education which should lay the foundations for life-long learning and be enjoyable, secure, and be based on a holistic approach to the needs of the children. Children in Sweden attend a 10-year compulsory school between ages 6–16 years (preschool class to year 9). Most Swedish children then continue to upper secondary school, sixth form or high school (years 10–12), although it is optional. Children with intellectual disability can attend special schools [24]. The Swedish Education Act [25] provides every child the right to have support to optimize their development and learning and achieve the knowledge requirements for each grade. There are two main categories of school-based support in the Swedish mainstream schools; “special” support and school-based accommodations. “Special” support is a part of the pupil’s action plan, has longer duration, includes greater interventions in the school which are not possible to conduct by the ordinary teachers/school staff and its implementation needs a formal decision from the principal. School-based accommodations refers to a less intensive support, which is provided by ordinary teachers and school staff, without the need for a formal decision by the principal [26]. In the Swedish compulsory school, 5.8% of the pupils had an action plan as a part of special school-based support in year 2021/2022 [27].

#### Children with LGEA

**Fig. 1:**
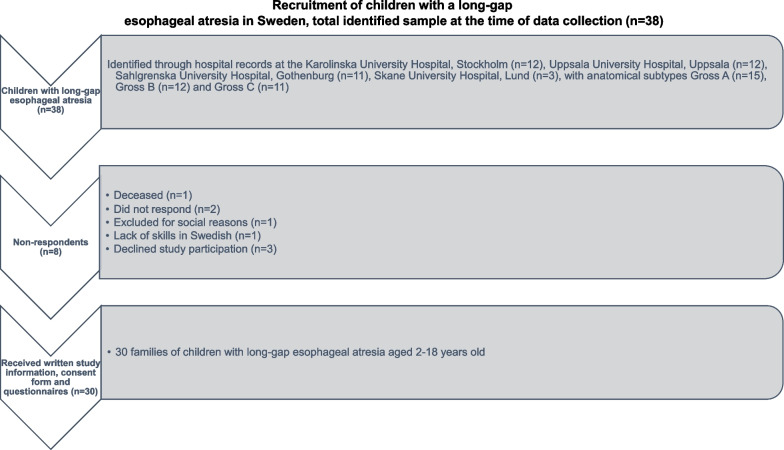
The recruitment of children with a long-gap esophageal atresia in Sweden, total identified sample at the time of data collection (n = 38)

Children were considered to have LGEA when primary anastomosis was not achievable at the first operation because it was too far between esophageal segments. They were recruited nationwide through collaboration with all four Swedish pediatric surgical centers, where the children were surgically treated and offered follow-up care. Inclusion criteria were child ages 2–18 years, respondents fluent in written and spoken Swedish, written informed consent by children aged ≥ 15 and legal guardians of all children. Exclusion criteria was delayed reconstruction of EA only due to prematurity/low birth weight. Out of 38 families of children with LGEA, 30 families received written study information, consent form and the questionnaires for this study (see Fig. 1). Data was collected from mid-January to March in 2020, then was paused due to the Covid-19 pandemic. The last four replies were collected between February and April in 2021.

#### Comparison group; children with EA Gross type C, who underwent primary anastomosis

Ninety-five children with Gross EA type C who underwent primary anastomosis (PA) at Sahlgrenska University Hospital, Gothenburg and participated in earlier studies of school support and school functioning (> 90% response rate) from 2017 to 2019 [17, 28, 29] served as an hypothesized milder affected comparison group for children with LGEA regarding available school data.

#### Clinical data

A researcher at each study center reviewed medical records for birth characteristics, anatomical subtype of EA, associated anomalies, surgical interventions, postoperative outcomes, including the latest anthropometric growth measurement with date for measurement, weight and height values, norm SD values for child age and sex. Information on the child’s airway and digestive problems the past four weeks was collected through a parent-reported questionnaire.

#### Parent/family characteristics

One parent of each child completed a survey asking about parental age, marital status and educational level.

#### School situation

One of the child’s parents answered a survey developed by the authors and which was based on the terminology/definitions for school-based support provided by the Swedish National Agency for Education [26], see Additional file 1. Information of the child’s school-functioning was assessed by using one of the four subscales from PedsQL™ 4.0 generic core scales (PedsQL™ 4.0) [30], which has been evaluated for use in healthy children and children with chronic conditions. In children aged 2–4 the domain school functioning comprises three items asking about problems with keeping up or performing like peers and missing school/nursery and in children > 4 years five items asking about problems with paying attention in school, forgetting things, keeping up with school activities and missing school. These questions are answered using a 4-week recall period and a 5-point Likert scale.

#### Mental health

Mental health was measured by the Strength and Difficulties Questionnaire (SDQ), a brief well validated screening instrument with sound psychometric properties for children in Sweden [31]. In this study, we used the parent-rated version, which encompasses 25 items allocated into five scales; emotional symptoms, conduct problem, hyperactivity/inattention, peer relationships and prosocial behavior. The Likert response scale range from “not true”, “somewhat true” or “certainly true”, is rated 0–2 for negatively worded items and inversely 2–0 for positively worded items.

### Data analysis

Statistical data were analyzed using IBM SPSS Statistics for Windows (version 25.0, Armonk, NY, USA: IBM Corp). The study sample characteristics of children/parents, school based-support, school absence, school functioning and school satisfaction were analyzed using descriptive statistics. Parents’ open answers were categorized according to their content; type of school support and reason for school absence and then analyzed using descriptive statistics. School absence ≥ 1 times/month the past year was regarded as high frequency [17, 32]. For continuous variables, median and range were calculated and for categorical variables, frequencies and percentages.

In accordance with the SDQ instrument instructions [33, 34], a total difficulties score (0–40) was calculated by summing up the four scales measuring emotional symptoms (0–10), conduct problems (0–10), hyperactivity/inattention (0–10), and peer relationship problems (0–10); higher scores indicate more problems. The subscale prosocial behavior is inverted; higher scores (0–10) represent better prosocial behavior. Cut-off values for ‘abnormal’ (≥ 90 percentile) provided in the Swedish User Support [33] were applied to evaluate the children’s level of mental health. A descriptive comparison of children with LGEA with reported abnormal levels of mental health and provision of school-based support was conducted.

Fisher’s exact test was applied to examine if the proportions for school-based support and school absence were different in children with LGEA vs PA and within subgroups of children with LGEA.

Spearman’s rank correlation coefficient (Spearman’s rho, r_s_) was used for bivariate correlation for school-based support and school absence respectively with each of the three numerical variables child age, presence of different digestive (heartburn, difficulties swallowing, vomiting problems) and respiratory symptoms (wheezing, cough, airway infections, chest tightness, dyspnea) the past month.

The responses to the 5-point Likert scale of PedsQL™ 4.0 were transformed to a 0–100 scale score, with higher levels reflecting better school-functioning [30]. The Mann–Whitney U-test was used to analyze if there were statistically significant differences in the school functioning scores between children with LGEA and PA.

In the group of children with LGEA, relationship between school functioning and school satisfaction respectively, with presence of school-based support and school absence were analyzed using r_s_.

We regarded r_s_ 0–0.39 as weak, 0.40–0.59 as moderate and ≥ 0.60 as strong correlations.

Significance level was considered at *p* < 0.05.

## Results

### Study population

Twenty-six families of children with LGEA returned signed consent forms and the questionnaires (87% response rate). The characteristics of the children and their parents vs the sample with PA are presented in Table 1. Child age or sex distribution did not differ between those with LGEA vs PA (*p* > 0.05).

**Table 1:** Characteristics of the study sample of children and adolescents with long-gap esophageal atresia (n = 26) and parents acting as proxy for their child vs those with primary anastomosis of esophageal atresia Gross type C (n = 95)

	Long-gap esophageal atresia, n (%)	Primary anastomosis , n (%)	*p* value
***Congenital/neonatal***			
Male sex	13 (50.0)	57 (60)	0.38
Prematurely born (< 37 gestational weeks)	15 (57.7)	31 (33)	0.039
Low birth weight (< 2500 g)	17 (65.4)	33 (35.9)	0.012
Gross type A	11 (42.3)		
Gross type B	8 (30.8)		
Gross type C	7 (26.9)	95 (100)	
Associated anomalies^a^	18 (69.2)	57 (60)	0.50
Cardio-vascular	8 (30.8)	26 (27.4)	0.81
Anorectal	7 (26.9)	10 (10.5)	0.052
Uro-genital	10 (38.5)	10 (10.5)	0.002
VACTERL^b^	6 (23.1)	17 (17.9)	0.56
Genetic disorder	4 (15.4)	10 (10.5)	0.50
Initital gap length, median cm (range)^c^	4 (2–7)		
Initital gap length, median vertebral bodies (range)	4 (2–6)		
***Surgery***			
Delayed primary anastomosis	11 (42.3)		
Esophageal replacement^d^	15 (57.7%)		
Anastomotic leakage	8 (30.8)	9 (9.6)^e^	0.011
Revisional surgery due to anastomotic leakage or recurrent fistula	4 (15.4)	9 (9.5)	0.47
***Symptoms the past four weeks***			
Swallowing difficulties	8 (30.8)	40 (42.6)	0.38
Heartburn	9 (34.6)	34 (36.6)	1.0
Vomiting problems	7 (26.9)	24 (25.5)	1.0
Cough	14 (53.8)	50 (52.6)	1.0
Wheezing	8 (32.0)	32 (34.0)	1.0
Chest tightness	9 (34.6)	13 (14.0)	0.023
Airway infections	10 (38.5)	27 (28.7)	0.35
Dyspnea at exercise or rest	9 (34.6)	49 (52.1)	0.13
***Treatment at follow-up***			
Gastrostomy feeding at follow-up	6 (23.1)	8 (8.4)	0.08
Antireflux surgery	9 (34.6)	12 (12.6)	0.017
Esophageal dilatation	20 (76.9)	38 (40.0)	< 0.001
Antireflux medication	18 (75.0)^e^	28 (29.5)	< 0.001
Inhaled steroids and/or bronchodilators	14 (58.3)^e^	39 (41.1)	0.17
Other medication	15 (57.7)	35 (36.8)	0.036
***Child age***			
Child age, median (range)	11 (3–17)	9 (2–17)	0.30
***Parent-proxy***			
Male sex	6 (23.1)	13 (13.7)	0.24
Age, median (range)	45 (33–58)	41 (26–69)^c^	0.064
Less than University/College degree	9 (34.6)	7 (7.3)	0.18
Living as single parent	2 (8.0)	15 (15.8)	0.52

^a^cardio-vascular, gastrointestinal, urogenital, limb, vertebrae-rib, choanalatresia, eye, ear, central nervous system or respiratory anomaly

^b^VACTERL stands for vertebral defects, anal atresia, cardiac defects, tracheo-esophageal fistula, renal anomalies, and limb abnormalities. Individuals diagnosed with VACTERL association have at least three of these characteristic features’

^c^1 missing value

^d^Gastric pull-up (n = 2), Partial gastric pull-up (n = 3), Gastric tube esophagoplasty preserving the distal esophageal segment (n = 8), colon interposition (n = 2)

^e^2 missing values

### School-based “special” support and accommodation

In total, 17 children with LGEA (65.4%) had either school-based “special” support and/or “accommodations”.

#### Prevalence and type

Table 2 presents the prevalence and type of “special” support in school among children with LGEA. As shown, 14(53.8%) of them had experienced “special” support and 11 (42.3%) received “special” support at the time of the study (median 1 type, range 0–4 of the listed types of support in Table 2). When parents described the children’s “special” support in own words, it revealed that the children needed help from school staff with special health care needs (n = 8) and educational support (n = 6).

**Table 2:** Prevalence and type of school-based “special” support in children with long-gap esophageal atresia aged 3–17 years

**School-based support (n = 26 replies)**	**n (%), long-gap EA**
Earlier or current school support	14(53.8)
Current school support	11(42.3)
Regular contact with special teacher	9(34.6)
Special teaching group	4(15.4)
Student assistant	9(34.6)
Other	7(26.9)
**School-based support: parents’ (n = 11) descriptions**	
***Support with health care needs (n = 8 children)***	
Assistants who help with daily medical management including medications, tube feeding and/or emptying of the bladder (n = 5)	
Prevention of or help with acute respiratory issues (n = 2)	
Room to rest (n = 1)	
***Educational support (n = 6 children)***	
Regular contact with a mentor (n = 1)	
Additional staff and pedagogical resource (n = 2)	
Shortened lessons (n = 1)	
Extra teaching support in math (n = 1)	
Extra support to improve concentration/focus (n = 2)	
Adjusted learning material/pictorial support (n = 1)	
Adjusted sport class (n = 1)	

Table 3 presents the prevalence and type of school-based accommodations in children with LGEA. Sixteen children with LGEA (64%) were either earlier or currently recipients of school-based accommodations and 14 (56%) utilized school accommodations at the time of the study (median 1 type, range 0–6 of the listed categories of school accommodation in Table 2). When their parents described the children’s school support in own words, they explained how the children were supported in their nutritional intake situation (n = 10 children). This included environmental accommodations, eating special food to prevent food impaction, choking or gastro-esophageal reflux symptoms as well as having an adult beside them in the school cafeteria to supervise, prevent food impaction and choking or assist at such events. Moreover, the parents gave details of their educational accommodations (n = 4 children), adjustment to manage their special needs (n = 4 children) and explained that their child received help to structure activities during the school day (n = 1 child).

**Table 3:** Prevalence and type of school-based accommodations in children with long-gap esophageal atresia aged 3–17 years

**School-based accommodations (n**_**tot**_ **= 25 replies)**	**n (%), long-gap EA**
Earlier or current school accommodation	16(64.0)
Current school accommodation	14(56.0)
Help to plan and structure a school day/schedule	5(20.0)
Extra clear instructions	5(20.0)
Adjusted learning materials	5(20.0)
A special teacher during a limited time of the day	9(36.0)
Support with nutritional intake issues	14(56.0)
Other	9(36)
**School-based accommodations: parents’ (n = 14) descriptions**	
***Nutritional intake support (n = 10 children):***	
Having an adult beside them in the school cafeteria to supervise, prevent food impaction and choking or assist at such events (n = 10)	
Environmental accommodations like a special place in the school cafeteria, eating in the classroom or close to a toilet in case of choking (n = 3)	
Special food intake to prevent food impaction, choking or gastro-esofageal reflux symptoms (n = 4)	
***Education adjustements (n = 4 children):***	
Activities during sport class (n = 1) or math (n = 1)	
Adjusted learning materials (n = 1)	
Scheduled breaks/rest (n = 1)	
Teaching assistant (n = 1)	
Special teacher (n = 1) or smaller peer groups (n = 1) during lessons or special school (n = 1)	
Help to structure the school day (n = 1)	
***Support with special health care needs (n = 4 children):***	
Managing of tube feeding (n = 2)	
Monitoring of growth and blood pressure once a month (n = 1)	
Supervision to prevent extraction of g-tube (n = 1)	

#### Lack of school-based support

As seen in Table 4, all children with LGEA who were reported abnormal levels on the SDQ scales had school-based support, except for one child with abnormal levels of hyperactivity/inattention. In the school survey, one parent reported a lack of school-based support and school accommodation for their child and needed to help their child in school on their own. Another parent currently reported the provision of support, but desired more for their child.

**Table 4:** Comparison of parent-reported abnormal scores on mental health using the Strengths and Difficulties questionnaire and use of either school-based “special” support or accommodation

Abnormal levels of the Strength and Difficulties questionnaire, parent-report	School-based “special” support or accommodation	
	Yes	No
Total difficulties (n = 5)	5	0
Emotional symptoms (n = 3)	3	0
Conduct problems (n = 4)	4	0
Hyperactivity/inattention (n = 7)	6	1
Peer relationships (n = 8)	8	0
Prosocial behaviour (n = 4)	4	0

#### Comparison between children with LGEA and PA

Table 5 presents the comparison of school-based “special” support and/or accommodations between children with LGEA and PA. As shown, the use of either “special” support and/or school accommodations was significantly more common in children with LGEA compared to children with PA (65.4 vs 36.8%, *p* = 0.013). When categorized into any intervention in school to assist the child’s nutritional intake/meals or education, it was observed that a significantly higher proportion of children with LGEA than children with PA had nutritional intake support in school (60% vs 20%, *p* < 0.001).

**Table 5:** Comparison of school-based support and/or accommodations between children with long-gap esophageal atresia (n = 26) and children with Gross type C esophageal atresia, primary anastomosis (n = 95) using Fisher’s exact test

	Long-gap esophageal atresia		Gross type C esophageal atresia, primary anastomosis		*p* value
	Yes	No	Yes	No	
School-based “special” support and/or accommodations	17 (65.4)	9 (34.6)	35 (36.8)	60 (63.2)	0.013
Educational intervention	13 (50.0)	13 (50.0)	28 (29.8)	66 (70.2)^a^	0.064
Nutritional intake support or accommodations	15 (60.0)^a^	10 (40.0)	19 (20.2)	75 (79.8)^a^	< 0.001
Special school for children with intellectual disability	1 (4.0)^a^	24 (96.0)	5 (5.3)	90 (94.7)^a^	1.0

^a^1 missing value

#### Associated factors

Additional file 2 presents the relationship between child characteristics and use of school-based support in children with LGEA. Their use of “special” support was associated with low birth weight (*p* = 0.036) and an increased number of different digestive symptoms (*p* = 0.033). Use of school-based accommodations was related to young child age (*p* = 0.014), an increased number of different digestive symptoms (*p* < 0.001), airway symptoms (*p* = 0.011) and height ≤ −2SD for age and sex (*p* = 0.024).

### School-absence

Table 6 details the frequency and reasons of school absence in children with LGEA and PA. Nine children with LGEA (36%) had high frequency of school absence (≥ 1 times/month) the past year, which was not significantly different from the proportion of children with PA (28.9%), *p* = 0.63. Colds/airway infections (*p* = 0.045) and GI-specific problems (*p* = 0.003) were significantly more frequently mentioned by parents with LGEA compared to PA as a reason for school absence. As shown in Additional file 3, relationship between child characteristics and school absence the past year, higher level of school absence in children with LGEA was related to young child age (*p* = 0.022), an increased number of different digestive symptoms (*p* = 0.012) and airway symptoms (< 0.001).

**Table 6:** Frequency and reasons of school absence in children with long-gap esophageal atresia (n_tot_ = 25) and with primary anastomosis of Gross type C esophageal atresia

School absence the past year	Long-gap esophageal atresia	Gross type C esophageal atresia, primary anastomosis	*p* values
School-absence	n, %	n, %	
High frequency of school absence^a,^ ^b^	9 (36.0)	26 (28.9)	0.63
Several times every month	7 (28.0)	17 (18.9)	
At least once a month	2 (8.0)	9 (10.0)	
3–5 times half a year	5 (20.0)	20 (22.2)	
3–5 times every year	7 (28.0)	31 (34.4)	
About once every year	4 (16.0)	4 (4.4)	
No school absence	0	9 (10.0)	

Reasons for school absence ^c,^ ^d^	n, %	n, %	
Colds/Airway infections	21 (87.5)	56 (65.1)	0.045
Repeated respiratory problems	6 (25.0)	21 (24.4)	1.0
Health care consumption	8 (33.3)	20 (23.3)	0.43
General sickness^e^	11 (45.8)	48 (55.8)	0.49
GI-specific problems^f^	8 (33.3)	7 (8.1)	0.003
Sleep disturbance/Tiredness	3 (12.5)	7 (8.1)	0.44
Other reasons^g^	2 (8.3)	9 (10.5)	1.0

^a^ ≥ 1 month/year

^b^ 3 missing values  in the group of children with Gross type C esophageal atresia, primary anastomosis

^c^ 2 missing values in the group of children with long-gap esophageal atresia

^d^ 7 missing values in the group of children with Gross type C esophageal atresia, primary anastomosis

^e^when parents described e.g. fever, headache, ill

^f^including vomiting problems, gastroesophageal reflux disease and stomachache

^g^when parents described non-disease related reasons like travels, menstruation in girls, visit at the dentist

### School satisfaction and school functioning

In children with LGEA, the median school functioning scale scores (n_tot_ = 25, median 75, range 15–100) were 10 points lower, but not significantly different from children with PA (n_tot_ = 89; median 85, range 18.8–100), *p* = 0.34. Their school-functioning scores, correlated negatively and strongly with “special” support (r_s_ = −0.62, < 0.001), school-based accommodations (r_s_ = −0.64, < 0.001) and school absence (r_s_ = −0.60, *p* = 0.002).

Parents of children with LGEA (n_tot_ = 25) reported their children’s school satisfaction as very good (n = 18, 72%), good (n = 2, 8%), relatively good (n = 4, 16%) and “not good” (n = 1, 4%). The rated school satisfaction of children with LGEA (n_tot_ = 25) had a significant negative relationship with “special” support (r_s_ = −0.47, *p* = 0.019), but not with school-based accommodation (r_s_ = −0.25, *p* = 0.24) or school absence (r_s_ = −0.38, *p* = 0.060), suggesting that use of “special” support in school was associated with less school satisfaction in children with LGEA.

## Discussion

This nationwide Swedish study is its first of its kind and revealed that 65% of children with LGEA utilized school-based support, reflecting their multifaceted daily needs in nutritional intake situations, education and medical management.

In this study, 42% of children with LGEA were reported to use “special” support in school, which was requisite of formal decision from the principal. “Special” support consisted of educational support to large extent. In comparison, 5.8% of the pupils in the Swedish compulsory school had an action plan as a part of “special” support in school[27]. Furthermore, the parents’ reports of their children’s school-based accommodations also reflected educational support. The proportion of children with LGEA who received educational support seems higher than in Swedish pupils in general and in earlier reports of children with EA which have been described that 22%-35% access special education[15–17]. In relation to other pediatric surgical malformations, 52% of children with anorectal malformations and 55% of children with Hirschsprung’s disease have been reported to receive special education and remedial teaching [35]. However, the definitions for “special” educational support and age of the study populations vary between studies [17].

Fifty-six percent of children with LGEA were recipients of school-based accommodations, all of whom had needed support regarding their nutrition intake. In our study, all children with LGEA and height ≤ 2SD for child age/sex at follow-up required this support. Growth retardation may be associated with weakened cognitive performance [36], in turn posing the need also for educational support. In this study, several children with LGEA needed school-based supports related to education/learning and nutritional intake. It is known that children with LGEA risk developing long-term esophageal complications [4, 8, 9, 37]. In agreement, we found more of them to having had antireflux treatments, esophageal dilations and nutritional intake support in school as opposed to children with PA who were expected to suffer from milder complications. This together implies that esophageal morbidity contributes to great care needs.

Furthermore, we observed that children with LGEA needed school-based support in relation to tube feeding and medical management, which can be provided based on the Swedish Act concerning Support and Service for Persons with Functional Impairments [38]. Parents may have described overlapping information to the questions of school-based “special” support and accommodation. Nevertheless, it together provides a more comprehensive understanding of their needs.

Special education in children with EA has been described to be more common in children with concomitant anomalies [17, 18], such as cardiovascular anomalies [19] or anorectal malformations [18]. In other studies, children with LGEA have been found to more commonly present with cardio-vascular malformations [5, 6] and genetic disorders [5] compared to short-gap EA with PA. However, in this study, prematurity and low birth weight, which are interrelated, were more commonly present. Low birth weight, even controlled for confounders, is associated with lower intelligence in the general population [39] and has earlier been shown to independently predict use of educational support in children with EA [17]. Moreover, the associated factors related to school-based support in children with LGEA were digestive and respiratory symptom burden, suggesting that there is a more disease burdened subgroup with multiple daily needs. In this study, use of school-based accommodations in children with LGEA was also related to young child age, but this was not found regarding “special” support. According to the Swedish definition, it could mean that children with LGEA at younger age needs less interventional support by ordinary school staff, which have not yet been included in an action plan with additional school resources and decided upon by the principal. However, school-based accommodations included support in nutritional intake issues in all cases. Previous research of coping abilities in children with EA [40] has shown that the child’s ability to deal with challenging nutritional intake situations increases with age, as their need for additional adult support at meals decreases. This could therefore help to explain our findings.

In this study, most parents reported that their child had received necessary school-based support and almost all children with LGEA with abnormal mental health according to SDQ were recipients of school-based support. A vulnerability in children with LGEA to develop mental health or behavioral problems probably reflects a complex interplay of congenital, clinical (e.g. exposure of anesthesia and surgery early and repeatedly in life, experience of somatic illness), nutritional and psychosocial factors [41], which should be considered in relation to their situation in school. However, in comparison to the known challenges children living with a rare disease may generally have in accessing inclusive and quality education [14, 42], our results could be viewed as encouraging. Perhaps this is attributable to the Swedish school systems/laws which provide these children right to school-based support [24–26]. The national follow-up care program for children with EA, does not yet formally include a standardized provision of psychological consultation to the child [43], but the pediatric surgeon can mandate the child to have school-based support, which seems to work adequately in most cases of children with LGEA in Sweden.

We found that 36% of children with LGEA were absent from school ≥ 1times/month the past year (equivalent to at least 12 times the past year). This concurs with previous studies showing that children with chronic conditions experience frequent periods of school absenteeism [44, 45]. In contrast to earlier studies suggesting school absenteeism to be related to disease severity [45], we found that high frequency of school absence did not differ between children with LGEA and PA. However, in agreement, children with LGEA were more frequently absent from school due to colds/airway infections and GI-specific morbidity. Both restrictive and obstructive lung dysfunction are observed as common in Swedish children with LGEA [12], which could contribute to these study findings. Respiratory morbidity is very common at young age [23, 37] and in this study school absence also correlated to young child age, suggesting an interrelationship. Aerodigestive morbidity may have different and interrelated aetiology. Airway and digestive symptoms may be related to gastroesophageal reflux, tracheomalacia, esophageal dysmotility and strictures [46]. It may originate from a disturbed development of the respiratory tract [47], but also as a result of surgical complications [46, 48]. Knowledge of school absence and its possible reasons in children with LGEA is important to recognize, as it has been described by parents of children with EA to impair social integration with peers and school achievement [17] and it is an independent factor that negatively influences family functioning [32].

We found that children with LGEA did not differ in scores of the school functioning domain in the PedsQL 4.0 compared to children with PA. This may be due to small sample size in LGEA, but it is also possible the provided school-based support has increased the level of school functioning. In comparison with other studies, children with chronic conditions have been found to functioning worse in school regarding peer relationships, academic achievement, and lack of engagement in school [44, 45, 49]. Previous research of children treated in advanced pediatric surgery has focused on academic achievement and found that a subgroup of children with gastroschisis (complex gastroschisis) SPS:refid::bib50[50], and children with congenital diafragmatic hernia (those with oxygen at discharge and longer initial hospital stay) perform worse academically [51]. In the whole group of children with EA, Burnett et al. [52] found a mixed picture regarding school functioning, with attention and working memory regarded as most vulnerable areas. Considering PedsQL 4.0 as a summative indicator of school functioning [30], we found that the subgroup of LGEA children with aerodigestive symptoms and those with school-based support risk poorer school functioning. It is possible that they need more intensive support in school than they currently receive. As LGEA is a congenital condition, it may be important to consider that children hospitalized for a chronic condition as early as during the preschool period are at risk of academic underperformance as they progress in school [53, 54].

Furthermore, most parents in this study rated their child’s school satisfaction as good, which was important as we earlier have found elevated levels of peer relationship problems in 31% of children with LGEA [20].

### Study limitations

The study is limited by the small sample, heterogeneity related to anatomical subtypes, gap length, associated anomalies and surgical methods as well as by the number of non-participants in LGEA. Gap length for definitions of LGEA is debated, but 76.1% of participants at the ERNICA consensus conference on LGEA agreed that it refers to a gap of 3 vertebral bodies or more [2]. In our study, only two children had 2 cm or 2 vertebral bodies at initial gap measurement. Furthermore, the study population has a broad age-span, incorporating data from preschool children to upper secondary school. However, given the small sample size despite our nationwide recruitment of children with LGEA, investigations into narrower age subgroups would not be statistically feasible. Similarly, if we had excluded children with LGEA and genetic disorders, the sample would become less statistically feasible. There was no statistical difference in the overall prevalence of genetic disorders or associated anomalies between LGEA and PA, and the parents could describe in the survey if their child attended a special school. In other Swedish studies, children with VACTERL (stands for Vertebral defects, Anal atresia, Cardiac defects, Tracheo-esophageal fistula, Renal anomalies, and Limb abnormalities. Individuals diagnosed with VACTERL association have at least three of these characteristic features) reported psychological well-being which was comparable to the norm group of Swedish school children [55] and intelligence was within the normal range [56]. However, attention difficulties were found in eight out of ten preschool children with VACTERL, requiring adjustments at school, and two of these were later diagnosed with attention deficit hyperactivity disorder [56]. The frequency and nature of associated anomalies should therefore also be considered in relation to our study findings. Moreover, there is no standardized definition of problematic school absence, and we defined a cutoff for high frequency of school absence which had been previously used [17, 32]. Although we complied with terminology utilized by the Swedish National Agency for Education [26], the school questions, apart from those in the PedsQL 4.0, were not part of a valid scoring instrument and we collected data from parents. This study did not provide information on LGEA children’s academic achievement, total amount of time absent from school or compared school data with a general control group. The data on PA children were collected during a different time period.

## Conclusions

This nationwide Swedish study revealed that 65% of children with LGEA utilized school-based support, reflecting multifaceted daily needs in their nutritional intake situations, education/learning and medical management. Overall school-based support was related to aerodigestive symptoms, “special” educational support specifically to low birth weight and school-based accommodations in nutritional intake situations to young child age and height ≤ −2SD at follow-up. School absence is frequent in children with LGEA and related to poorer school functioning. The study findings are important for health care providers, families and patient support groups for EA as they can help inform and prepare families of children with EA what needs children with EA/LGEA may have in everyday life. Given that the rights/access to school support may vary between countries, this study could also be used as first template on the goal of creating equality on the rights to support in school when living with a rare disease such as LGEA/EA. Such a goal could be included in the stakeholders’ recommendations for a holistic care and treatment in children with EA. For health care providers especially, the findings highlight the need to accommodate disease-specific needs, including those that become apparent in school. In clinical practice, this could include provision of standardized and holistic follow-up care of children with LGEA, including both multidisciplinary monitoring and treatment of aerodigestive disease, evaluation how the children function in school and regard their quality of life. Future research should identify the schooling experiences of children with LGEA in other countries, facilitators and barriers for these children to receive necessary support, assess the neurocognitive functioning, learning and academic achievement of children with EA, as well as their educational and employment level in adulthood and satisfaction with professional choice in life.

### Supplementary Information

**Additional file 1.** Presentation of the school survey.

**Additional file 2.** Relationship between child characteristics and school-based “special” support. Relationship between child characteristics and school-based “accommodation”.

**Additional file 3.** Relationship between child characteristics and school absence the past year.

## Data Availability

The datasets analyzed during the current study are available in the manuscript or in its additonal files. Further information is not available in public due to lack of ethical approval.

## Abbreviations

EA: Esophageal atresia
LGEA: Long-gap esophageal atresia
PA: Primary anastomosis
PedsQL 4.0™: Pediatric quality of life inventory
