# Supplementary material for: Schooling experiences in children with long-gap esophageal atresia compared with children with esophageal atresia and primary anastomosis: a Swedish study
Source: Orphanet J Rare Dis. 2023 Aug 7;18:233. doi: 10.1186/s13023-023-02846-8 (PMC10408199; doi:10.1186/s13023-023-02846-8)
Supplement: Supplementary file 1 — Additional file 1. Presentation of the school survey. [file 13023_2023_2846_MOESM1_ESM.docx]

| Supplemental material 1 Presentation of the school survey | | |
| --- | --- | --- |
| *The school should provide the necessary ”special” support and accommodations for all pupils who need school-based support. We have the following questions regarding your child’s school situation.* | | |
| **“Special” support** |  |  |
| *“Special” support includes greater interventions in the school which are most often long-lasting. These should be part of the pupil’s action plan which describes the support the child and its implementation needs a formal decision from the principal* |  |  |
| Has your child received school-based ”special” support? | - Yes | - No |
| Does your child currently have school-based ”special” support? | - Yes | - No |
| If YES, does the ”special” support include | - Yes | - No |
| - Regular contact with special teacher | - Yes | - No |
| - Special teaching group | - Yes | - No |
| - Student assistant | - Yes | - No |
| - Other | - Yes | - No |
| Describe with your own words what the school-based ”special” support include | | |
| If NO, do you wish to have school-based ”special” support for your child | - Yes | - No |
| **School-based accommodations** |  |  |
| *School-based accommodations refers to a less intensive support, which is normally provided by ordinary teachers, school staff and within ordinary class/educational resources.* |  |  |
| Has your child received school-based accommodations? | - Yes | - No |
| Does your child currently receive school-based accommodations? | - Yes | - No |
| If YES, do these school-based accommodations include | - Yes | - No |
| - Help to plan and structure a school day/schedule | - Yes | - No |
| - Extra clear instructions | - Yes | - No |
| - Adjusted learning materials | - Yes | - No |
| - A special teacher during a limited time of the day | - Yes | - No |
| - Support with nutritional intake issues | - Yes | - No |
| - Other | - Yes | - No |
| Describe with your own words what the school-based accommodations include | | |
| If NO, do you wish to have school-based accommodations support for your child? | - Yes | - No |
| **School-satisfaction** |  |  |
| How do you experience your child’s school satisfaction?   - Very good (5) - Good (4) - Relatively good (3) - Not good (2) - Not good at all (1) | | |
| Any further comments regarding your child’s school satisfaction | | |
| **School- absence** | | |
| Please estimate, how often your child was absent from school the past year?   - Several times each month - 1 time/month - 6-11 times/year - 3-5 times/year - Around 1 time the last year - No absence from school | | |
| If you child was absent from school the past year, which were the reasons? Please list the reasons for school absence and describe with your own words  List of reasons for school absence:  1)  2)  3)  4) | | |
