# Supplementary material for: Schooling experiences in children with long-gap esophageal atresia compared with children with esophageal atresia and primary anastomosis: a Swedish study
Source: Orphanet J Rare Dis. 2023 Aug 7;18:233. doi: 10.1186/s13023-023-02846-8 (PMC10408199; doi:10.1186/s13023-023-02846-8)
Supplement: Supplementary file 2 — Additional file 2. Relationship between child characteristics and school-based “special” support. Relationship between child characteristics and school-based “accommodation”. [file 13023_2023_2846_MOESM2_ESM.docx]

| Supplemental material 2. Relationship between child characteristics and school-based “special” support | | | | |
| --- | --- | --- | --- | --- |
|  | **Definition** | **School-based ”special” support** | | **p-value** |
| **Categorial factors** |  | Yes | No |  |
| Child sex | male | 5(38.5) | 8(61.5) | 1.0 |
|  | female | 6(46.2) | 7(53.8) |  |
| Gestational age at birth | prematurity (< 37 weeks) | 8(53.3) | 7(46.7) | 0.25 |
|  | normal gestational age at birth | 3(27.3) | 8(72.7) |  |
| Birth weight | low birth weight (< 2500 grams) | 10(58.8) | 7(41.2) | 0.036 |
|  | birth weight (≥2500 grams) | 1(11.1) | 8(88.9) |  |
| VACTERL^a^ | VACTERL, yes | 3(50.0) | 3(50.0) | 1.0 |
|  | VACTERL, no | 8(40.0) | 12(60.0) |  |
| Weight ≤-2SD for age | Yes | 5(55.6) | 4(44.4) | 0.43 |
|  | No | 6(37.5) | 10(62.5) |  |
| Height ≤-2SD for age | Yes | 5(71.4) | 2(28.6) | 0.18 |
|  | No | 6(33.3) | 12(66.7) |  |
| **Numerical factors** |  | Spearman’s rho | |  |
| Child age years | (range 3-17) | -0.16 | | 0.58 |
| Airway symptoms^b^ | (range 1-5) | 0.31 | | 0.13 |
| Digestive symptoms^c^ | (range 1-3) | 0.42 | | 0.033 |
| ^a^ VACTERL stands for vertebral defects, anal atresia, cardiac defects, tracheo-esophageal fistula, renal anomalies, and limb abnormalities. Individuals diagnosed with VACTERL association have at least three of these characteristic features  ^b^ Airway infections, Cough, Dyspnea, Wheezing, Chest tightness  ^c^ Swallowing difficulties, Heartburn, Vomiting problems | | | | |

| Supplemental material 2. Relationship between child characteristics and school-based “accommodation | | | | |
| --- | --- | --- | --- | --- |
|  | **Definition** | **School-based accommodations** | | **p-value** |
| **Categorial factors** |  | Yes | No |  |
| Child sex | male | 7(53.8) | 6(46.2) | 1.0 |
|  | female | 7(53.8) | 5(41.7) |  |
| Gestational age at birth | prematurity (< 37 weeks) | 7(50.0) | 7(50.0) | 0.69 |
|  | normal gestational age at birth | 7(63.6) | 4(36.4) |  |
| Birth weight | low birth weight (< 2500 grams) | 11(68.8) | 5(31.3) | 0.16 |
|  | birth weight (≥2500 grams) | 3(33.3) | 6(66.7) |  |
| VACTERL^a^ | VACTERL, yes | 5(83.3) | 1(16.7) | 0.18 |
|  | VACTERL, no | 9(47.4) | 10(52.6) |  |
| Weight ≤-2SD for age | Yes | 5(62.5) | 3(37.5) | 1.0 |
|  | No | 9(56.3) | 7(43.8) |  |
| Height ≤-2SD for age | Yes | 6(100) | 0 | 0.024 |
|  | No | 8(44.2) | 10(55.6) |  |
| **Numerical factors** |  | Spearman’s rho | |  |
| Child age years | (range 3-17) | -0.48 | | 0.014 |
| Airway symptoms^b^ | (range 1-5) | 0.51 | | 0.011 |
| Digestive symptoms^c^ | (range 1-3) | 0.64 | | <0.001 |
| ^a^ VACTERL stands for vertebral defects, anal atresia, cardiac defects, tracheo-esophageal fistula, renal anomalies, and limb abnormalities. Individuals diagnosed with VACTERL association have at least three of these characteristic features  ^b^ Airway infections, Cough, Dyspnea, Wheezing, Chest tightness, max n=5  ^c^ Swallowing difficulties, Heartburn, Vomiting problems, max n=3 | | | | |
