# Supplementary material for: Schooling experiences in children with long-gap esophageal atresia compared with children with esophageal atresia and primary anastomosis: a Swedish study
Source: Orphanet J Rare Dis. 2023 Aug 7;18:233. doi: 10.1186/s13023-023-02846-8 (PMC10408199; doi:10.1186/s13023-023-02846-8)
Supplement: Supplementary file 3 — Additional file 3. Relationship between child characteristics and school absence the past year. [file 13023_2023_2846_MOESM3_ESM.docx]

| Supplemental material 3. Relationship between child characteristics and school absence the past year | | | | |
| --- | --- | --- | --- | --- |
|  | **Definition** | **School absence ≥ 1 times/month the past year** | | **p-value** |
| **Categorial factors** |  | Yes | No |  |
| Child sex | male | 5(38.5) | 8(61.5) | 1.0 |
|  | female | 4(33.3) | 8(66.7) |  |
| Gestational age at birth | prematurity (< 37 weeks) | 4(28.6) | 10(71.4) | 0.43 |
|  | normal gestational age at birth | 5(45.5) | 6(54.6) |  |
| Birth weight | low birth weight (< 2500 grams) | 6(37.5) | 10(62.5) | 1.0 |
|  | birth weight (≥2500 grams) | 3(33.3) | 6(66.7) |  |
| VACTERL^a^ | VACTERL, yes | 4(66.7) | 2(33.3) | 0.14 |
|  | VACTERL, no | 5(26.3) | 14(73-7) |  |
| **Numerical factors** |  | Spearman’s rho | |  |
| Child age years | (range 3-17) | -0.46 | | 0.022 |
| Airway symptoms^b^ | (range 1-5) | 0.75 | | <0.001 |
| Digestive symptoms^c^ | (range 1-3) | 0.49 | | 0.012 |
| ^a^ VACTERL stands for vertebral defects, anal atresia, cardiac defects, tracheo-esophageal fistula, renal anomalies, and limb abnormalities. Individuals diagnosed with VACTERL association have at least three of these characteristic features  ^b^ Airway infections, Cough, Dyspnea, Wheezing, Chest tightness, max n=5  ^c^ Swallowing difficulties, Heartburn, Vomiting problems, max n=5 | | | | |
